# Prolonged Altered Mental Status in a Diabetic Hemodialysis Patient

**DOI:** 10.7759/cureus.13132

**Published:** 2021-02-04

**Authors:** Mojgan Jalalzadeh, Ashok Chaudhari, Donald Baumstein

**Affiliations:** 1 Internal Medicine/Nephrology, Metropolitan Hospital Center, New York Medical College, New York, USA

**Keywords:** type 1 diabetes mellitus, hyperosmolar hyperglycemic state, osmotic demyelination syndrome, end-stage renal disease (esrd)

## Abstract

Osmotic demyelination syndrome (ODS) is a demyelinating disorder of the central nervous system. It usually occurs with rapid correction of severe chronic hyponatremia. ODS is rarely seen as a complication of hyperglycemia. Herein, we report a rare presentation of ODS secondary to hyperosmolar hyperglycemic state. A 28-year-old female with type 1 diabetes, hypertension, seizure disorder, and end-stage renal disease on hemodialysis was brought from a shelter with two days of unresponsiveness and developed ODS after hyperosmolar hyperglycemic state in long-standing uncontrolled diabetes with normal serum electrolyte levels.

## Introduction

Osmotic demyelination syndrome (ODS), the term used for both central pontine myelinolysis and extrapontine myelinolysis [1], is a demyelinating disorder of the central nervous system. It was first described in 1959 by Adams et al. in alcoholic and malnourished patients [2]. It is a rare but life-threatening neurological disorder, which is commonly noted with the rapid correction of severe hyponatremia [3] but infrequently reported from other scenarios of rapid changes of plasma osmolality [4]. Other medical conditions such as liver disease, hypokalemia, hypophosphatemia, hypoglycemia, and folate deficiency are associated with ODS [3].

## Case presentation

A 28-year-old female with a history of end-stage renal disease on hemodialysis (HD), type 1 diabetes mellitus (DM), hypertension, seizure disorder, non-compliant with therapy, was brought to the Emergency Department for recent gait abnormality followed by unresponsiveness for two days and one missed dialysis session. Recently, she had a history of multiple admissions for hyperglycemia and hypoglycemia. One week prior to this admission, she was admitted to MICU with serum glucose of 752 mg/dL, Na of 130 mEq/L, K of 5.3 mEq/L, bicarbonate of 21 mEq/L, anion gap of 12, lactate of 1 mmol/L, negative serum ketones, and normal liver function tests. On presentation, she was afebrile with blood pressure of 175/99 mmHg, and Glass Coma Score (GCS) was 6 out of 15 (opens eyes spontaneously, no verbal response, no motor response, grimaces in response to pain).

**Figure 1:**
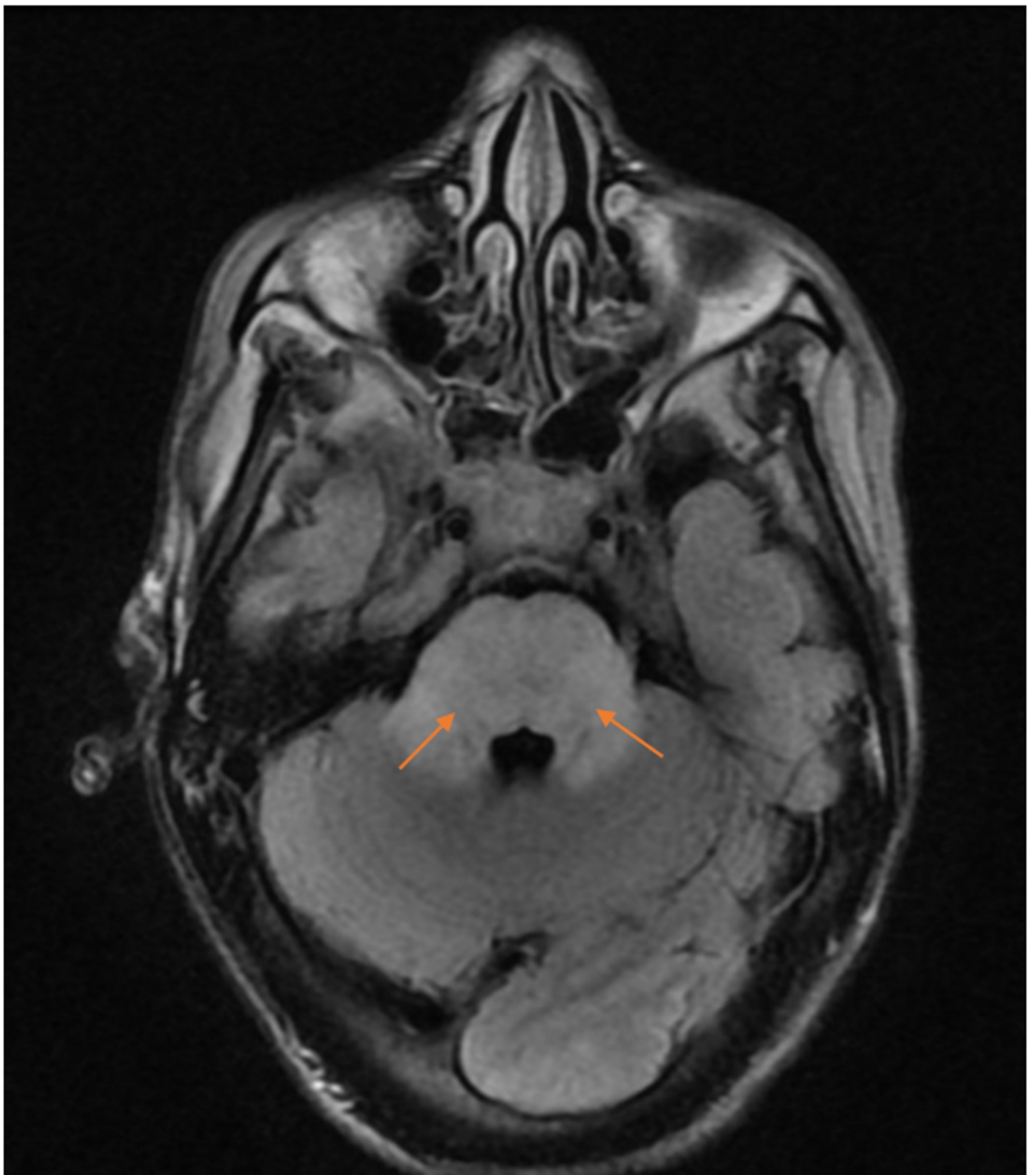
T2 MRI image of the brain showing multifocal T2 hyperintensity within the white matter bilaterally in a fairly symmetric distribution (arrows). Extensive T2-hyperintensity within the midbrain and pons involving the cerebellar peduncles is seen due to demyelinating processes.

**Figure 2:**
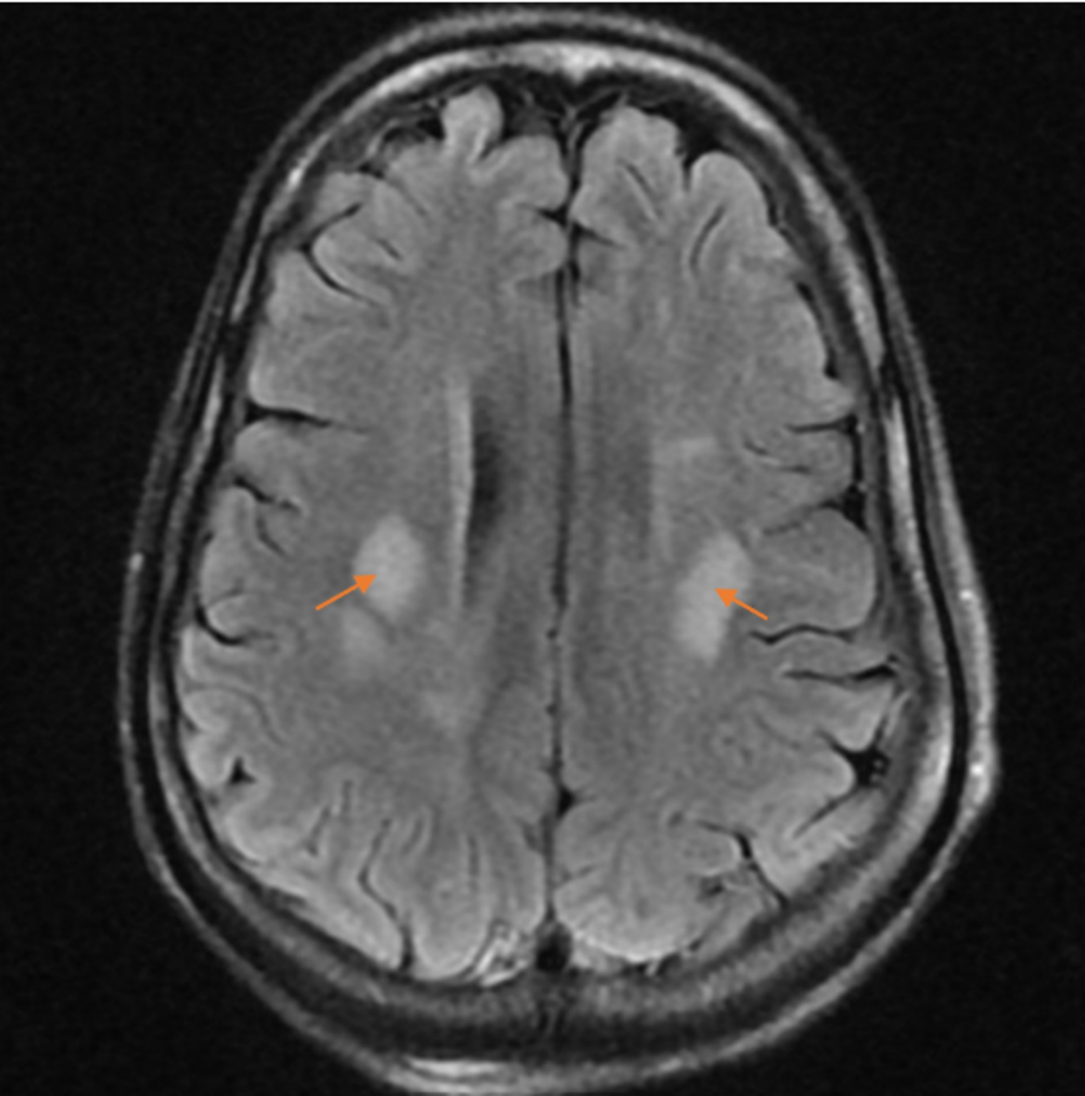
T2 MRI image of the brain showing areas of restricted diffusion within the deep white matter at the bilateral frontoparietal junctions (arrows). In this setting, the restricted diffusion could relate to active demyelination. Active demyelination is suspected.

Labs were notable for Glu of 105 mg/dL, Na of 135 mEq/L, K of 4.9 mEq/L, Cl of 102 mEq/L, bicarbonate of 22 mEq/L, anion gap of 16, blood urea nitrogen of 69 mg/dL, creatinine of 7.7 mg/dL, magnesium of 2.5 mg/dL, calcium of 8 mg/dL, phosphorous of 4.2 mg/dL, venous pH of 7.37, and lactate of 0.6 mmol/L, normal liver function tests normal, subtherapeutic phenytoin levels (<4 µg/mL), urine toxicology negative for phencyclidine (PCP) and tetrahydrocannabinol (THC), salicylate level < 1.7 mg/dL, acetaminophen < 2 µg/mL, ammonia of 7 µmol/L, HIV, negative JC polyomavirus polymerase chain reaction (PCR) of cerebrospinal fluid (CSF), negative herpes simplex virus type 1 and type 2 PCR of CSF, negative blood and CSF bacterial cultures, negative fungal blood culture, and negative B-Hcg (beta-human chorionic gonadotropin). Non-contrast brain CT showed mild cerebral edema. She received urgent HD and was intubated for status epilepticus. Her blood pressure stabilized, but her GCS was unchanged. EEG showed disorganized slow background due to metabolic encephalopathy. Brain MRIs obtained are shown in Figures 1, 2.

She developed aspiration pneumonia and intermittent fevers. On transthoracic echo, no vegetations were found. She underwent tracheostomy and percutaneous endoscopic gastrostomy (PEG) placement and continued with antiepileptics. Since her mental status was not improving, a trial of pulse intravenous methylprednisolone was given without yielding any benefit. Her mental status did not improve for the two-month duration of her hospitalization, and she subsequently died after transfer to a nursing home.

## Discussion

In hyponatremic states, brain cells can regulate osmolality by reducing fluid absorption and cerebral swelling [5]. Initially, fluid, sodium, and potassium move from the cerebral interstitial space into the CSF. Then in hours to days, the amount of osmolytes (myoinositol, glutamine, taurine) is reduced from inside the brain cells [6]. In hypernatremia states, regulatory mechanisms increase the level of osmolytes inside the brain cells, which reduces the loss of fluids to the extracellular space [7].

During rapid correction of sodium, ODS occurs due to water being driven out of the brain’s cells to compensate for increased extracellular tonicity, and there is no time for adjustment of osmolytes inside of brain cells [5]. Similarly, the hypertonic insult of severe acute hyperglycemia may occur faster than the rate at which brain cells can compensate leading to ODS. This leads to disruption of the blood-brain barrier and entrance of cytokines and immunoglobulins to the central nervous system, which damage the oligodendrocytes and myelin [1]. Clinical manifestations of ODS include dysarthria, dysphagia, paraparesis or quadriplegia, altered mental status, seizure, and, in severe cases, locked-in syndrome that usually occurs five to seven days after the onset of hypertonic status, but they may also appear after two or more weeks [8].

Hyperosmolar hyperglycemic state (HHS) in patients with type 1 DM associated with ODS is rare and is rarely diagnosed, with few case reports in the literature [5,9,10]. Our patient had a long-standing poorly controlled type 1 DM without electrolyte imbalance, and the symptoms of ODS developed gradually. ODS associated with glucose derangements has been reported in the following conditions: long-standing poorly controlled blood sugar, concomitant with or without electrolyte change, and occurrence after treatment of HHS [4, 5,9]. Hepatitis C, hypertension, and diabetic nephropathy requiring dialysis are considered as risk factors for ODS in diabetic patients [11].

In ODS, the MRI typically reveals non-contrast enhancing symmetric hyperintense lesions on T2-weighted images, as seen in Figures 1, 2, as noted by the radiologist. The radiologist additionally noted that the bilateral, nearly symmetric distribution of pathology in this case is suggestive of metabolic disease, toxin exposure, or demyelinating process.

The differential diagnosis for ODS includes stroke, encephalitis, meningitis, Wernicke encephalopathy, hepatic encephalopathy, primary brain tumors, metastases, radiotherapy, chemotherapy, and multiple sclerosis [12]. All these conditions were excluded in this case. There is no specific treatment for non-sodium-dependent ODS, and management is largely supportive [13]. However, it is important that the hypertonic condition should be lowered gradually. In some ODS cases, steroid bolus, intravenous immunoglobulin, and thyrotropin-releasing hormone had been used [14,15]. The outcome of ODS varies from complete recovery to death or permanent disability [16].

The clinical outcome of in this case was poor. Because of ODS and the consequent mental status change, our patient required intubation and long-term mechanical ventilation. This set her on the course of further complications, which we believe led to her death.

## Conclusions

Diagnosis of ODS requires a high index of suspicion and is usually caused by overly rapid correction of hyponatremia. HHS in a long-standing uncontrolled DM is a rare cause of ODS. This occurs days after acute hyperosmolar insult, as in our case. Clinicians should consider ODS in diabetic patients with a history of poorly controlled blood glucose and new neurologic symptoms with consideration of brain MRI to assist in this diagnosis.
